# Oxysterols as Reliable Markers of Quality and Safety in Cholesterol Containing Food Ingredients and Products

**DOI:** 10.3389/fnut.2022.853460

**Published:** 2022-02-16

**Authors:** Federico Canzoneri, Valerio Leoni, Ginevra Rosso, Davide Risso, Roberto Menta, Giuseppe Poli

**Affiliations:** ^1^Soremartec Italia Srl, Ferrero Group, Alba, Italy; ^2^Laboratory of Clinical Chemistry, ASST Brianza, School of Medicine and Surgery, Hospital of Desio, University of Milano Bicocca, Milan, Italy; ^3^Unit of General Pathology and Physiopathology, Department of Clinical and Biological Sciences, San Luigi Hospital, University of Turin, Turin, Italy

**Keywords:** cholesterol, oxysterols, COPS, autoxidation, cytotoxicity, food processing, shelf life, markers

## Abstract

Cholesterol is a lipid of high nutritional value that easily undergoes oxidation through enzymatic and non-enzymatic pathways, leading to a wide variety of cholesterol oxidation products (COPs), more commonly named oxysterols. The major oxysterols found in animal products are 7α-hydroxycholesterol, 7β-hydroxycholesterol, 7-ketocholesterol, 5α,6α-epoxycholesterol, 5β,6β-epoxycholesterol, cholestan-3β,5α,6β-triol, and 25-hydroxycholesterol. They are all produced by cholesterol autoxidation, thus belonging to the non-enzymatic oxysterol subfamily, even if 7α-hydroxycholesterol and 25-hydroxycholesterol are, in part, generated enzymatically as well. A further oxysterol of the full enzymatic origin has recently been detected for the first time in milk of both human and bovine origin, namely 27-hydroxycholesterol. Nowadays, gas or liquid chromatography combined to mass spectrometry allows to measure all these oxysterols accurately in raw and in industrially processed food. While non-enzymatic oxysterols often exhibited *in vitro* relevant cytotoxicity, above all 7β-hydroxycholesterol and 7-ketocholesterol, 27-hydroxycholesterol, as well as 25-hydroxycholesterol, shows a broad spectrum *in vitro* antiviral activity, inhibition of SARS-CoV-2 included, and might contribute to innate immunity. Quantification of oxysterols was afforded over the years, almost always focused on a few family's compounds. More comprehensive COPs measurements, also including oxysterols of enzymatic origin, are, nowadays, available, which better display the many advantages of systematically adopting this family of compounds as markers of quality, safety, and nutritional value in the selection of ingredients in processing and storage. Regarding foodstuff shelf life, COPs monitoring already provided useful hints for more suitable packaging. The identification of a subset of non-enzymatic and enzymatic oxysterols to be routinely assessed in food production and storage is proposed.

## Introduction

Cholesterol is a lipid of high nutritional value that easily undergoes oxidation, both enzymatically and not enzymatically driven, by this way leading to a wide variety of cholesterol oxidation products (COPs), more commonly named oxysterols. Oxysterols differ from the parental molecule for an epoxy- or hydroxyl- or ketone group in the steroid nucleus or a hydroxy group in the hydrophobic side chain (1, 2). In all animals, humans included, widely distributed specific enzymes represent an important endogenous source of oxysterols, of which those of main physiological interest are 27-hydroxycholesterol [systematic name (25R) 26-hydroxycholesterol] (27OHC), 24-hydroxycholesterol (24OHC), 7α-hydroxycholesterol (7αOHC), 20α-hydroxycholesterol (also 20S-hydroxycholesterol) (20αOHC), 25-hydroxycholesterol (25OHC) (3–5). Of note, the last three oxysterols, namely 7αOHC, 20αOHC, and 25OHC, may be, in part, generated non-enzymatically as well (3, 5).

**Figure 1:**
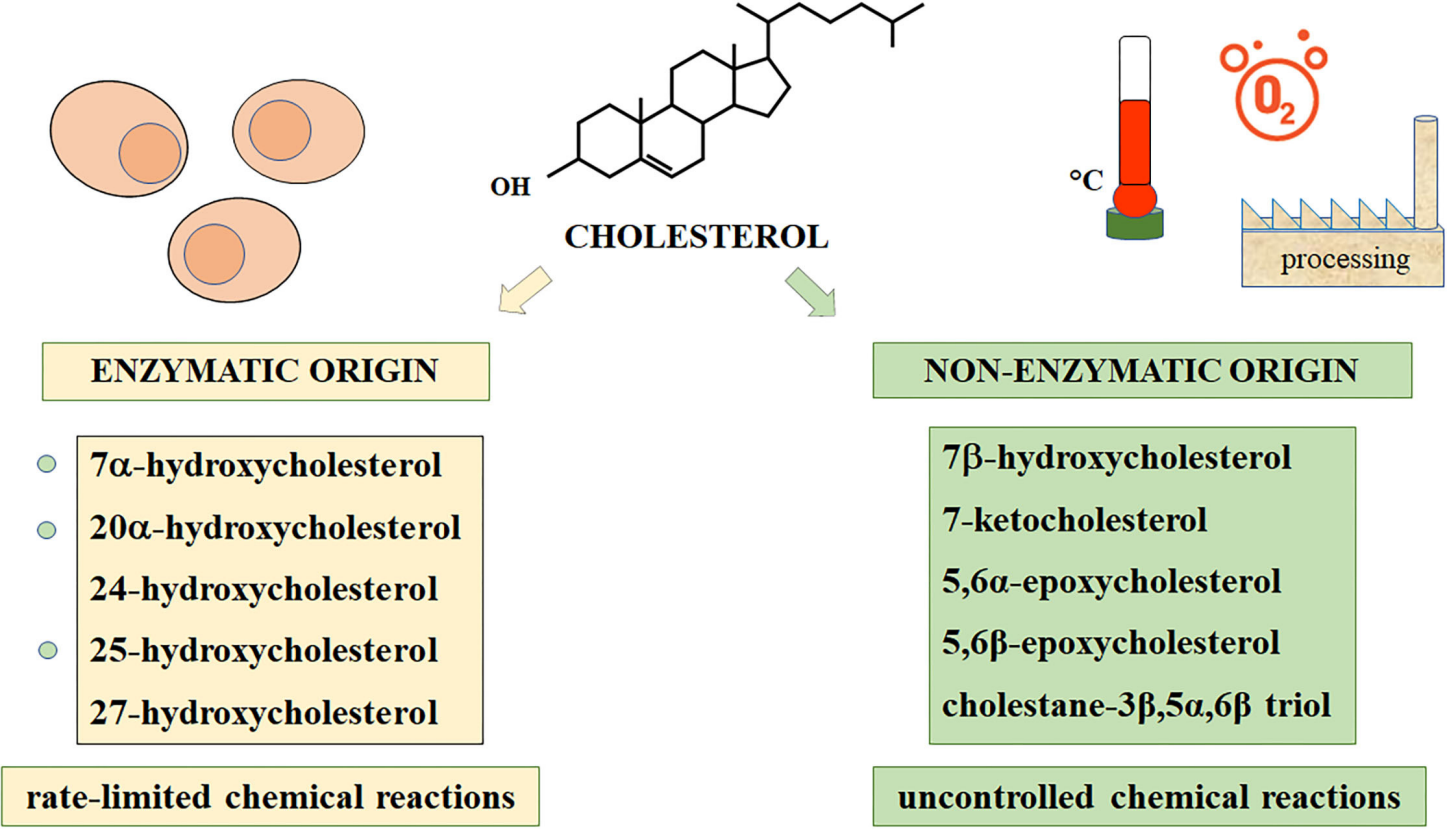
Main oxysterols of enzymatic and non-enzymatic origins.

Indeed, cholesterol autoxidation can be induced in food ingredients and products by heat, light exposure, refrigeration, freeze-drying, and spray-drying, leading to the formation of non-enzymatic oxysterols, namely 7β-hydroxycholesterol (7βOHC), 7-ketocholesterol (7KC), 5α,6α-epoxycholesterol (α-epoxy), 5β,6β-epoxycholesterol (β-epoxy), cholestan-3β,5α,6β-triol (triol) (3, 5, 6). Another important source of the same non-enzymatic cholesterol oxides, in this case an endogenous one, is represented by the reactive oxygen species (ROS) that are generated in a variety of pathophysiological conditions in tissues and cells; indeed, high amounts of oxysterols are produced during the inflammatory process that often sustains and promotes chronic human diseases (7, 8). In Figure 1, all main enzymatic and non-enzymatic oxysterols of pathophysiological interest are reported.

With regard to the detection and quantification of oxysterols in biological samples, as well as in food, mass spectrometry either coupled to gas chromatography (GC-MS) or liquid chromatography (LC-MS/MS) has been adopted for many years. However, because of the relatively very low amount of oxysterols as to cholesterol in the various matrices, the difficult chromatographic separation of some of these compounds, and the easy autoxidation of cholesterol during the extraction and purification procedures, often generated data that are lacking in precision and are hardly comparable. Only very recently, consensually standardized GC-MS and LC-MS/MS procedures have finally allowed to obtain accurate, precise, and reliable quantification of oxysterols from different sources (2, 9).

Hereafter, the measurement of oxysterols in food industry will be reviewed and discussed, with some emphasis on the cutting-edge results achieved on milk and milk-derived products. For an adequate and complete analysis of the high translational power of oxysterols quantification in food and other industry fields, see also another recent review by Poli et al. (10).

## Measurement of Oxysterols in Food Industry: General Aspects

Many reports have been published over the years, providing quantification of oxysterols of a non-enzymatic origin in chicken, pork or beef meat, fish, egg, butter, whole or skimmed milk powder [for a review see (6, 11)]. Because of the above reported complexity of oxysterols measurement, the collected data were not always comparable to each other. In addition, quite often, only a few oxysterols were analyzed, and, rarely, an actually comprehensive detection was carried out, despite the frequent use of the COPs term. Anyway, even if not all non-enzymatic oxysterols of pathophysiological interest were searched and detected, 7αOHC, 7βOHC, and 7KC consistently appeared as those quantitatively more represented in the different foods examined, their concentration was significantly varying, for example, with the total cholesterol, total polyunsaturated fatty acids, and total antioxidants content of a given food or food product.

Only very recently, the concentration of oxysterols in foodstuff has been analyzed in a more comprehensive way, in particular in milk, milk powder, and related products, including chocolate (12–14). Certainly, such a more complete approach took advantage from the technology, meanwhile much improved and standardized, and, at least, in part, suggested by the previous analyses afforded on human breast milk. Indeed, all major oxysterols of both enzymatic and non-enzymatic generation were detectable in human colostrum, intermediate and mature milk (9). Of particular interest was in this case the novel observation of a high concentration of 27OHC in the colostrum, which, later on, confirmed to occur in bovine colostrum as well (12). 27OHC, named galactosterol since among the different cholesterol-containing foods, which was shown for the first time in the (cow) milk, has recently been shown, along with 25OHC to have strong and wide-spectrum antiviral properties [for a review see (10, 15)].

The antiviral potential of 27OHC, present in mature bovine milk in concentrations of physiological interest (12), as well as that of 25OHC, appears to be an emerging nutritional factor that could be considered in the overall evaluation of the quality of cholesterol-containing foods. Indeed, even if never investigated so far, 27OHC is certainly present in detectable amounts also in meat, fish, egg, and the other foods of animal origin.

## Potential Cytotoxicity of Non-Enzymatic Oxysterols in Food

While oxysterols of enzymatic or partially enzymatic generation, like 27OHC and 25OHC, respectively, have shown remarkable broad antiviral effects, only toxicologic effects have been described so far for non-enzymatic oxysterols when present in excess in the human body but also in the ingested food. By far, the two widely most abundant and more cytotoxic oxysterols formed by autoxidation are 7βOHC and 7KC. In a comprehensive review by (16), the toxicity of an excess amount of these two oxysterols was deeply analyzed with regard to the different body districts, including heart, brain, and gut, as observed both in *in vitro* cell lines and in *in vivo* animal models. These authors provided interesting and informative insights into the signaling mechanisms through which these two cholesterol oxides may induce oxiapoptophagy, a type of cell death associated with oxidative stress, apoptosis, and enhanced autophagy, and promote inflammation. Indeed, these and other oxysterols, when present in the body in excessive amount, could contribute, through the above-described mechanisms, especially through inflammation, to the expression and complication of major chronic diseases, namely cardiovascular, respiratory, intestinal diseases, cancer, diabetes (8, 17, 18). Last but not least, the tissue damage exerted by toxic oxysterols could interfere with healthy aging (19, 20).

Thus, the two oxysterols, 7βOHC and 7KC, this time in a mixture of cholesterol oxides, which are actually that obtainable by heating pure cholesterol for 3 h at 180°C (21), showed to be still the key players in exerting oxidative stress, inflammation, and, eventually, apoptotic death in *in vitro* cultures of differentiated intestinal cells of human origin, namely CaCo-2 cells (22, 23). A similar pathophysiologically relevant mixture of non-enzymatic oxysterols was then employed to challenge *in vitro* differentiated enterocyte-like CaCo-2 cells and its effect on the intestinal barrier permeability evaluated by measuring cell monolayer transepithelial electrical resistance (TEER), as well as the cellular level of the main components of the intercellular tight junctions, namely junctional adhesion molecule-A (JAM), occludin, and zonula occludens-1 (ZO-1). A marked decrease of the protein level of all three tested junction proteins, especially of ZO-1, was observed in such an *in vitro* experimental model (24). Of note, in the latter oxysterol mixture mimicking that occurring in a high cholesterol diet, the percent amount of 7KC was around 40%, and that of 7βOHC was nearly 15%; thus the two oxysterols together appeared likely responsible for at least half of the observed enterocyte tight junctions derangement (24).

Unfortunately, although dietary oxysterols might represent a risk for human health, no toxicity limit for such compounds has been specified yet. Cardenia and colleagues (11) suggested that the threshold of toxicological concern (TTC) (25) for unclassified compounds (.15 μg per single compound/person/day) should be utilized as reference. Probably, this recommendation is too careful. The toxicological studies carried on so far in the *in vitro* model represented by enterocyte-like cell lines suggest that the daily uptake of total oxysterols should not exceed 1-μM concentration (24), which would approximately correspond to 0.4 μg/g of product. Indeed, the amounts of total oxysterols of a non-enzymatic source very recently calculated on fresh whole milk and fresh egg powders, obtained by spray drying, (14) or in prototype milk chocolate tablets up to 120 days of shelf life (13), were confirmed to be within such a potentially acceptable threshold. Certainly, more accurate and reliable warning would be provided by a comprehensive pharmacokinetic analysis of the most dangerous non-enzymatic oxysterols administered to experimental animals individually or in a mixture. The few data presently available would indicate a better intestinal absorption of 7βOHC, 7KC, and α-epoxy in comparison to that of β-epoxy and 25OHC (26), with a relatively more rapid hepatic metabolism of 7KC as to 7βOHC (27).

## Further Generation of Non-Enzymatic Oxysterols During Food Processing and Storage

The accurate quantification of oxysterols, especially the autoxidation ones, in the raw food and food ingredients containing cholesterol, as well as in processed and stored foodstuff, definitely appears as a convenient procedure that should be systematically carried out. It would be a further valid tool to assess at the same time quality and safety of both ingredients and food products.

Cholesterol present in food is an essential nutrient for appropriate cell membrane structure, steroid hormones synthesis, numerous tissue functions of its metabolites, but it must be consumed in an adequate daily amount. In addition, its predisposition to undergo oxidation reactions deserves great attention. For these reasons, its concentration in a given food or food ingredient should be fine-tuned, but, afterwards, during processing and storage, its expected partial autoxidation should be monitored and adequately limited.

The spray drying of milk and eggs (14, 28), or the heat processing of meat (29) significantly increases the preexisting amount of potentially harmful cholesterol oxides. Fresh and pasteurized products, milk and eggs, contain lower COPs than the previous ones (30). Hence, the monitoring of at least the most represented and more toxic oxysterols, namely 7βOHC and 7KC, during food processing would allow to improve and better standardize the procedure itself.

The storage of food ingredients is another critical factor that could heavily modify the relative oxysterols concentration. Undoubtedly, the ultimate concentration of oxysterols in food products placed on the market depends very much on the time length and condition of the storage, whether in air or under a vacuum, on the packaging system, on the presence or absence of endogenous or supplemented antioxidants and type of fats (e.g., saturated or unsaturated) (11, 31).

In general terms, the storage/shelf life of food ingredients and final products seems, by far, the most critical phase. Chudy and Makowska (32) monitored by capillary gas chromatography (GC) the content of 7αOHC, 7βOHC, 7KC, α-epoxy, and β-epoxy in milk, egg, and dairy-egg powders, packaged in air atmosphere or under a vacuum, fresh or stored for various periods of time up to 24 months. A shelf life of 3 months already led to a significantly increased production of the five oxysterols in all examined food powders, while their rise almost halved in samples identical but packaged under a vacuum. Notable was the observation of striking quenching of both single and total oxysterols generation, either in air or in a vacuum, when the milk and egg powders were mixed, probably due to a competition for oxygen by the different autoxidation pathways (32). In a subsequent study by Chudy and Teichert, expanding the literature on the quantification of oxysterols in these commodities, reconfirmed the trend found in the previous study (14).

Storages under air or under increased oxygen percentage (32%) were compared in raw beef slices wrapped in a transparent shrink film and refrigerated. Total oxysterols increased with the time, in significant higher percent in the samples stored at 32% oxygen tension, being 7βOHC and 7KC, the quantitatively more represented cholesterol oxides (33). The same group further analyzed the effect of different types of packaging on the storage-induced increase of oxysterols. Frozen horse meat slices were wrapped with either transparent or non-transparent to light films and then stored at 0–4°C and exposed to light for 8 h. The protection against cholesterol photooxidation by wrapping in non-transparent to light film was remarkable indeed, with 7βOHC and 7KC still representing the main non-enzymatic oxysterols (34). Worth noting is the total oxysterols concentration measured at the end of the meat storage in the packaging protecting against light oxidation appeared significantly lower than that measured in identical samples at time-zero storage. The oxysterols that showed much higher reduction were α-epoxy and β-epoxy (34), and one likely explanation would be their faster reaction with protein amino groups, preferably lysine and histidine, in a process known as protein sterylation, actually occurring in food processing (35). One more reason is to prefer 7βOHC and 7KC as quality markers in a cholesterol-containing food industry and, at the same time, an incentive to further investigate in the near future the reactivity of oxysterols with proteins.

The effect of food storage on the concentration of these two oxysterols was also observed in commercial fish meals, with 7βOHC showing to be much more sensitive to autoxidation than 7KC (36). Interestingly, the same evidence was consistently obtained when measuring oxysterols content in milk powders (12) and in prototype milk chocolate tablets made with whole milk powder stored for different periods of time at controlled temperatures (20 ± 2°C) and humidity (<65% relative humidity) in the dark under non-vacuum conditions (13).

## Advantages of a Systematic Adoption of COPs Quantification in Cholesterol-Containing Food Ingredients and Final Products

According to the literature on oxysterols measurement in food industry available, and on the clear enhancing effect of food processing and storage on their original concentration, it appears plausible and even appropriate to systematically add oxysterols quantification to the main parameters presently used to check and maintain the quality of food of an animal origin during processing and storage (37). In Table 1-A, the main advantages of adopting oxysterols as markers of quality but also of nutritional value in the case of cholesterol-containing food are summarized. No doubt that measuring the relative amount of oxysterols in food ingredients from different suppliers would contribute to properly choose the most reliable ones. Moreover, oxysterols monitoring during food processing and packaging would allow to control their correct performance and even to improve them.

**Table 1:** Oxysterols as quality markers in food industry.

**A) Advantages**	**B) Oxysterols suitable to routinary monitor the quality of food ingredients and products**
° Selection of raw material suppliers	*7βOHC and 7KC*
° innovative science-based support of food freshness and quality	° The first two fully non-enzymatic oxysterols stemming from cholesterol autoxidation
° Improvement of industrial processing	° Relatively stable compounds
° Improvement of food product storage (shelf life)	° Same catabolic route
° Development of new packaging procedures (e.g., under vacuum or modified atmosphere)	° Already known in the literature
° Keeping low the level of oxysterols of toxicological concern	° Relatively high cytotoxicity in cell cultures (7βOHC being the most toxic non-enzymatic oxysterol)
° Promotion of emerging nutritional factors (e.g., oxysterols with antiviral or pro-osteogenic properties)	*27OHC*
	° Enzymatic origin only
	° Stable compound
	° The most represented side-chain oxysterol in human and cow milk
	° Molecule of high nutritional perspective

However, some oxysterols present in food could also have a relevant nutritional value. In fact, at least, some chain oxysterols of exclusive or partial enzymatic origin, like 27OHC and 25OHC, respectively, showed quite promising antiviral properties with a wide spectrum of action (10, 15). Both cholesterol metabolites could play a role in innate and adaptive immunity by contributing to fight not only viral but also bacterial infections through the depletion/segregation of accessible cholesterol by acting as chemoattractants of different immune cells and modulating macrophage differentiation and activity [for a review see (38)]. Moreover, 20α- or 20S-hydroxycholesterol, an oxysterol of both enzymatic and non-enzymatic sources, very recently detected and quantified in egg and milk powders (14), is considered to be provided with pro-osteogenic and anti-adipogenic properties [see (15)].

Until today, enzymatic oxysterols have not been much considered in food industry, even if their presence in raw material of an animal origin should have been expected, most likely because their nutritional potential has been disclosed just recently. A future development of enzymatic oxysterols monitoring in food industry is thus expected.

## Application of a Selected Subset of Oxysterols to Routine Monitoring of Food Quality

There are still numerous gaps to be filled with regard to oxysterols as has been highlighted by Garcia-Llatas et al. and Kilvington et al., starting from their quantification in foodstuff to the one of the most desirable, dietary intakes (37, 39). A comprehensive analysis of COPs is highly recommended but difficult to be applied in a routine way, and not simply for economic reasons. A more applicable solution to routinely check the commercial and nutritional value of food products would appear to be the monitoring of a restricted subset of oxysterols. As reported in Table 1-B, such a restricted selection that cannot miss 7βOHC, apparently, the best marker of cholesterol autoxidation and, concurrently, the most harmful among its oxidative metabolites. It is usually tested in combination of 7KC, another pretty harmful oxysterol of a non-enzymatic source. Convenient is to include a side chain oxysterol as well to potentially able to add a nutritional value to a given food product, which is 27OHC or 25OHC, being the first one of an enzymatic origin only and present in human and cow milk in concentrations of biological relevance (9). Of interest, milk processing, but above all, milk powder storage, moderately reduced the original content of 27OHC while significantly increasing that of 25OHC, being prone to autoxidation (12, 13).

## Oxysterols Toxicity and Medical Bioremediation

Preventing or at least quenching the formation of oxysterols in food production and cooking is certainly a must to guarantee safe food consumption. The group of Lizard adequately reviewed the most practical approach to quench or even prevent the formation of 7βOHC and 7KC, namely the addition of especially natural molecules provided with marked antioxidant effect, which are numerous, indeed (40) Tocopherols (α- and γ-tocopherols), oleic acid, polyphenols, and theobromine have been demonstrated to be some of the most effective compounds able to prevent the impair damages induced by 7KC. α-tocopherols, for instance, exert the most efficient cytoprotective effect, as well as prevention, in a dose-dependent manner, of 7KC-induced oxiapoptophagy and peroxisomal dysfunctions. Some of these molecules are also cytoprotective against 7βOHC (40). Thus, these data suggest that eating habits and food-industry *ad hoc* formulations rich in functional foods with such beneficial effect can have a role in the prevention of oxysterols-affected diseases.

Recently, another very innovative approach related to medical bioremediation, which could be combined to the food addition with suitable molecules able to reduce oxysterols autoxidation, has been the use of exogenous microbial enzymes for the degradation of oxysterols. In two studies conducted by Ghosh and Khare (41, 42), using two strains, specifically *Rhodococcus erythropolis* MTCC 3951 and *Pseudomonas aeruginosa* PseA, are able to strongly degrade 7KC, counteracting its cytotoxic effect. As mentioned by the authors, these microbial enzymes involved in the bioprocess, such as cholesterol oxidase, lipase, dehydrogenase, and reductase, could be used for protective purposes, in those food products that have undergone heat treatment, resulting in induced oxidative stress, minimizing the risk of possible harmful effects.

## Conclusions

Really comprehensive oxysterols measurements in food ingredients and products have finally become available only recently, allowing to clearly outline the various advantages to systematically adopt their quantification at the different stages of food industry, from the raw material up to the storage of the commercial product. Oxysterols definitely appear as suitable markers of quality, safety, and nutritional value in the selection of food ingredients in the most appropriate food processing and storage.

Still, a few current research gaps can be envisaged, which should be tackled in the near future: (1) reliable knowledge of the actual harmfulness of certain non-enzymatic oxysterols when present in mixture in foodstuffs; (2) the identification of those oxysterols that are actually able to react with aminoacid residues of proteins (protein sterylation) and the entity of such a reaction; (3) the elucidation of the bioavailability of oxysterols (absorption, distribution, metabolism, and disposition); (4) the interaction of food oxysterols with the gut microbiota; (5) the nutritional and/or medical remediation of oxysterols toxicity.

## Author Contributions

Conceptualization was contributed by DR, GP, and RM. Methodology was contributed by VL. Writing–original draft preparation was contributed by GP. Writing–review and editing were contributed by DR, FC, and GR. Supervision was contributed by VL and RM. All authors contributed to the article and approved the submitted version.

## Conflict of Interest

FC, GR, and RM are employed by Soremartec Italia Srl, Alba (CN, Italy). At the time of conceptualization, DR was employed by Soremartec Italia Srl, while his current employer is Tate and Lyle Italy SpA. GP and VL have scientific consultancy contracts with Soremartec Italia Srl.

## Publisher's Note

All claims expressed in this article are solely those of the authors and do not necessarily represent those of their affiliated organizations, or those of the publisher, the editors and the reviewers. Any product that may be evaluated in this article, or claim that may be made by its manufacturer, is not guaranteed or endorsed by the publisher.
